# Sensitive and Stable NCF/GO/Au@Ag SERS Substrate for Trace Detection of Polycyclic Aromatic Hydrocarbons

**DOI:** 10.3390/polym17121716

**Published:** 2025-06-19

**Authors:** Lili Kong, Xinna Yu, Qifang Sun, Meizhen Huang, Tianyuan Liu, Jie Chen

**Affiliations:** 1 School of Mathematics, Physics and Statistics, Shanghai University of Engineering Science, Shanghai 201620, China; lilikco@163.com; 2 School of Electronic Information and Electrical Engineering, Shanghai Jiao Tong University, Shanghai 200240, China; xinnayu@sjtu.edu.cn (X.Y.); mzhuang@sjtu.edu.cn (M.H.); 3 College of Information, Mechanical and Electrical Engineering, Shanghai Normal University, Shanghai 200234, China; qifangsun@shnu.edu.cn

**Keywords:** surface-enhanced Raman spectroscopy (SERS), localized surface plasmon resonance (LSPR), charge transfer, nanocellulose (NCF), polycyclic aromatic hydrocarbons (PAHs)

## Abstract

Polycyclic aromatic hydrocarbons (PAHs) have attracted significant attention due to their severe threats to both ecological systems and human health. In this paper, a high-performance surface-enhanced Raman spectroscopy (SERS) substrate based on NCF/GO/Au@Ag nanocomposites was developed, which enabled sensitive and stable detection of PAHs. The NCF/GO/Au@Ag substrate synergistic utilizes the localized surface plasmon resonance (LSPR) effect of Au@Ag core–shell nanorods and the additional interfacial charge transfer provided by graphene oxide (GO) to exhibit extremely high sensitivity. And the three-dimensional fibrous network of nanocellulose (NCF) improved nanoparticle dispersion uniformity. Combined finite element simulations and experimental studies verified that the dual plasmonic resonances (512 nm and 772 nm) of Au@Ag nanorods optimally match 785 nm excitation, yielding an enhancement factor of 5.21 × 10^5^. GO integration enhanced Raman signals by 1.68-fold through interfacial charge transfer, while the introduction of NCF reduced the signal relative standard deviation (RSD) from 36.88% to 4.29%. The NCF/GO/Au@Ag substrate achieved a detection limit of 10 μg/L for PAHs, demonstrating exceptional sensitivity and reproducibility.

## 1. Introduction

Polycyclic aromatic hydrocarbons (PAHs) represent a critical category of persistent organic pollutants that have attracted significant attention due to their severe threats to both ecological systems and human health [1,2]. Traditional analytical methods such as gas chromatography [3], gas chromatography–mass spectrometer [4], and high-performance liquid chromatography [5] for the detection of PAHs are usually accurate and sensitive, but they are expensive, time-consuming, and require sophisticated sample pretreatment.

Surface-enhanced Raman spectroscopy (SERS) is considered to be a sensitive, specific, and reliable analytical technique that can comprehensively display the molecular information of analytes [6,7] and has been widely applied in environmental monitoring and other fields [8,9,10]. Benefitting from the localized surface plasmon resonance (LSPR) in noble metal nanostructures, which is called the “hot spot”, the Raman signals of molecules close to the nanostructure is significantly enhanced [11,12,13]. Au/Ag nanoparticle colloids serve as effective SERS substrates, where precise control over nanoparticle size and morphology optimizes their LSPR properties [14,15], significantly enhancing the detection sensitivity for PAHs [16,17,18]. The electromagnetic “hot spot” generated by these substrates could amplify target molecular Raman signals by 10^4^–10^6^-fold [19,20]. However, liquid colloidal SERS substrates exhibit practical limitations such as the poor uniformity caused by nanoparticle aggregation and coffee-ring effect.

Compared to liquid-phase SERS substrates, solid-state SERS substrates demonstrate superior stability due to the supporting matrix that effectively prevents noble metal nanoparticle aggregation and performance degradation [21,22]. Specifically, periodic nanostructures fabricated on rigid substrates (e.g., silicon wafers or glass) via laser ablation or magnetron sputtering exhibit exceptional uniformity and batch-to-batch reproducibility [23,24]. However, current fabrication limitations constrain their detection sensitivity. For PAH trace analysis, solid substrates typically show limits of detection 1–2 orders of magnitude higher than their optimized liquid-phase counterparts [25]. In recent years, researchers have employed various strategies including structural optimization, material doping, and process improvement to enhance SERS substrate performance [26,27,28]. However, few substrates have achieved satisfactory and well-balanced performance across all critical metrics.

To address the challenge of simultaneously achieving high sensitivity and uniformity in SERS substrates, this study innovatively developed an NCF/GO/Au@Ag composite substrate, enabling sensitive and stable detection of PAHs. The Au@Ag core–shell nanorod structures introduce graphene oxide (GO) as a charge transfer bridge, synergistically utilizing both electromagnetic enhancement and chemical enhancement to improve substrate sensitivity. Additionally, the three-dimensional fibrous network of nanocellulose (NCF) immobilizes the nanostructures, enhancing substrate uniformity and ensuring signal stability. This substrate achieves a detection limit of 10 μg/L for PAHs, combining the high sensitivity of traditional liquid-phase substrates with the excellent stability of solid-phase substrates, providing a novel solution for environmental trace pollutant monitoring.

## 2. Materials and Methods

### 2.1. Materials

Ascorbic acid (C_6_H_8_O_6_, AA), silver nitrate (AgNO_3_), sodium hydroxide (NaOH), sodium borohydride (NaBH_4_) and hydrogen tetrachloroaurate (HAuCl_4_) were purchased from Lingfeng Chemical Co., Ltd. (Shanghai, China). GO films with a thickness of 1~2 nm were purchased from InnoChem Co., Ltd. (Beijing, China). Hexadecyl trimethyl ammonium bromide (C19H42BrN, CTAB), Rhodamine 6G (R6G), pyrene, and acenaphthylene powders were purchased from Aladdin Chemical Co., Ltd. (Shanghai, China).

### 2.2. Fabrication of NCF/GO/Au@Ag Substrate

Fabrication of Au@Ag nanorods: Au nanorods were synthesized by a seed-mediated growth method using AA as a reducing agent [29]. The silver-coated Au nanorod process was carried out via the silver nitrate reduction method according to Liu’s approach [30]. The obtained Au@Ag nanorod colloid was washed with deionized water and centrifuged (10,000 rpm for 10 min) for three times to remove excess chemical substances. Purified Au@Ag nanorod colloid was kept in tubes for further use.

Assembly of NCF/GO/Au@Ag substrate: 1 mL of GO aqueous solution (0.01 mg/ mL) and 500 μL of NCF hydrogel were added to 3 mL of purified Au@Ag nanorod colloid and sonicated 3 min for thorough mixing. Then, the NCF/GO/Au@Ag substrate was prepared.

### 2.3. Sample Preparation and SERS Detection

Preparation of simulated PAH solutions: First, PAH solids were dissolved in ethanol to prepare standard stock solutions. The stock solutions were then serially diluted to obtain aliquots with concentrations of 10 μg/L and 10^3^ μg/L, which were directly used for SERS measurements.

SERS detection: A 20 μL aliquot of the NCF/GO/Au@Ag jellylike substrate was dispensed onto an aluminum plate. Subsequently, 10 μL of the prepared PAH solution was dropped onto the substrate and allowed to incubate for several minutes. SERS spectra were recorded by the Hx-spec system under a laser of 100 mW and 785 nm, with an integration time of 3 s.

### 2.4. Apparatus and Measurements

A scanning electron microscope (JSM-7800F, JEOL, Tokyo, Japan) was used for morphological analysis. The SERS spectra were recorded by a self-developed portable Raman system Hx-spec. This Raman system was equipped with a 785 nm diode laser source (0~300 mW laser power), a smart probe and a compact Czerny-Turner spectrometer with a resolution of 7 cm^−1^ in spectral range of 200–2700 cm^−1^.

### 2.5. FEM Simulation and Data Analysis

The LSPR and electromagnetic enhancement properties of noble metal nanostructures were investigated using the finite element method (FEM). The dielectric constants of Au and Ag were defined according to the Drude model [31]. In the simulation setup, the nanostructures were dispersed in water (refractive index = 1.33) and illuminated by a linearly polarized plane wave with the electric field vector aligned along the x-axis.

The fifth-order polynomial fitting algorithm was used to subtract the background fluorescence. Then, the spectrum was normalized to the integrated area under the curve. The enhanced factor (EF) was calculated according to the following formula:

(1) $$\mathrm{EF}=\frac{I_{\mathrm{SERS}}}{I_{\mathrm{NOR}}}\cdot \frac{N_{\mathrm{SERS}}}{N_{\mathrm{NOR}}}$$

where $I_{\mathrm{SERS}}$ and $I_{\mathrm{NOR}}$ are the intensity of the spectra at 612 cm^−1^ enhanced by the NCF/GO/Au@Ag substrate and blank aluminum plate. $N_{\mathrm{SERS}}$ and $N_{\mathrm{NOR}}$ represent the number of R6G molecules excited by Raman scattering light. The limit of detection (LOD) was calculated according to the following equation:

(2) $$LOD=\frac{3\delta }{k}$$

where δ is the standard deviation of blank measurements, and k represents the slope of the linear regression equation. Relative standard deviation (RSD) was applied to evaluate the uniformity of NCF/GO/Au@Ag substrate:

(3) $$\mathrm{RSD}=\sqrt{\frac{\sum_{i=1}^{n}{\left(I_{i}-\bar{I}\right)}^{2}}{n-1}}/\bar{I}$$

where n represents the number of samples, $I_{i}$ is the intensity of the R6G spectra at 612 cm^−1^ of the *i*-th sample, and $\bar{I}$ is the average intensity of the n samples at 612 cm^−1^.

## 3. Results and Discussion

### 3.1. Characterization of NCF/GO/Au@Ag Substrate

**Figure 1:**
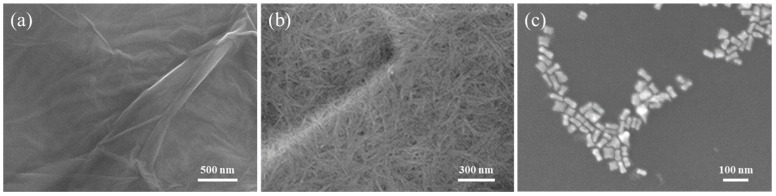
SEM characterization of NCF/GO/Au@Ag substrate. (**a**) GO, (**b**) NCF and (**c**) Au@Ag nanorods.

Figure 1a presents the SEM image of GO. GO exhibits a two-dimensional wavy membrane structure, where the corrugated wrinkles could provide abundant anchoring sites for nanostructures to form dense “hot spots”. As shown in Figure 1b, the NCF substrate consists of numerous microfilaments with diameters of several tens of nanometers and lengths up to the micrometer scale. These interwoven cellulose microfilaments construct a three-dimensional scaffold characterized by a high specific surface area and porosity. Figure 1c displays the Au@Ag core–shell nanorods, which exhibit a rod-like morphology with an aspect ratio of approximately 3.

### 3.2. LSPR Characteristics of Noble Metal Nanostructures

**Figure 2:**
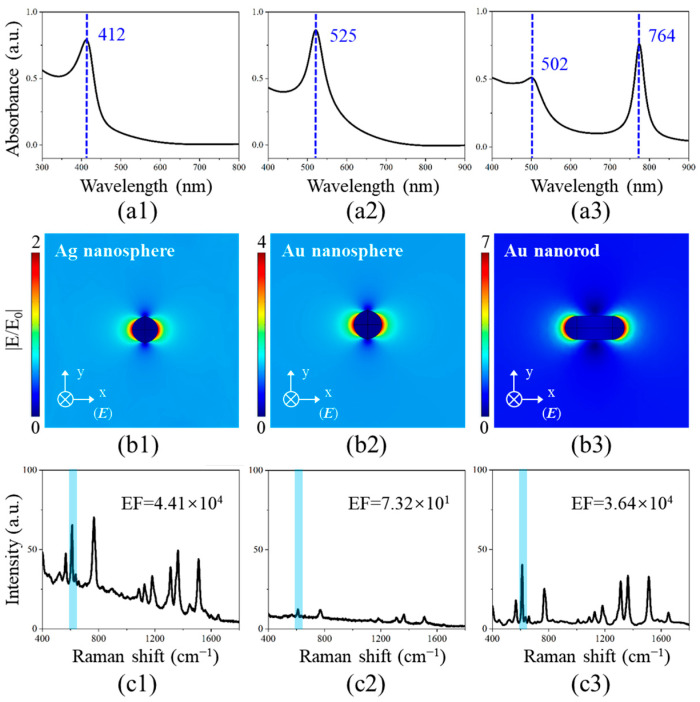
LSPR characteristics of noble metal nanostructures. (**a1**–**a3**) Simulated absorption spectra, (**b1**–**b3**) electric field distributions (785 nm excitation), and (**c1**–**c3**) SERS sensitivity test for Ag nanospheres, Au nanospheres, and Au nanorods. The characteristic peaks of 612 cm^−1^ are marked in blue.

Within the visible wavelength range, the distinctive dielectric properties of Au and Ag nanoparticles contribute to their larger scattering cross-sections, establishing them as the most widely utilized materials for SERS substrates. Among these, Au and Ag nanospheres are particularly prevalent due to their straightforward synthesis methods, while the anisotropic nature of Au enables symmetry breaking during growth to form rod-shaped structures. To investigate the LSPR characteristics of these metallic nanostructures, FEM simulations were employed to model Ag nanospheres, Au nanospheres, and Au nanorods, with the corresponding LSPR analysis presented in Figure 2.

Figure 2(a1–a3) presents the simulated absorption spectra of Ag nanospheres, Au nanospheres, and Au nanorods. Distinct LSPR peaks appear at 412 nm for Ag nanospheres and 525 nm for Au nanospheres, while Au nanorods exhibit two characteristic LSPR peaks near 502 nm and 764 nm, corresponding to transverse and longitudinal plasmon modes, respectively. These results demonstrate the shape-dependent plasmonic behavior of metal nanoparticles, in agreement with Mie and Gans theories [32,33,34]. Resonant excitation at the LSPR frequency induces collective plasmon oscillations, creating enhanced local fields. Figure 2(b1–b3) displays the electric field distributions of the Ag nanosphere, Au nanosphere, and Au nanorod under 785 nm excitation. The dual plasmon modes in Au nanorods significantly improve photon coupling efficiency, resulting in stronger localized fields. In contrast, Ag and Au nanospheres show relatively weaker electric field enhancement due to the spectral mismatch between the excitation wavelength and their resonance peaks. Experimental Raman measurements reveal that Ag colloidal substrates exhibit significantly stronger SERS activity compared to Au counterparts, attributable to their higher surface reactivity. As demonstrated in Figure 2(c1–c3) using R6G solution (10^−6^ M) as the probe molecule, the Ag colloidal substrate generates markedly enhanced Raman signals under identical measurement conditions. These results demonstrate that the electromagnetic field enhancement of noble metal nanoparticles under excitation laser exhibits strong dependence on both morphological characteristics and material composition. Specifically, the transverse and longitudinal plasmon resonance modes of Au nanorods enable broader spectral matching with incident laser wavelengths, while the superior surface activity of Ag nanoparticles contributes to their enhanced Raman response.

**Figure 3:**
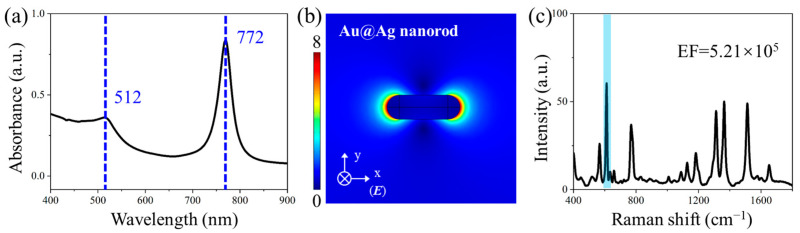
LSPR characteristics of Au@Ag nanorod. (**a**) Simulated absorption spectra, (**b**) electric field distributions (785 nm excitation), and (**c**) SERS sensitivity test, and the characteristic peak of 612 cm^−1^ is marked in blue.

Based on the aforementioned research results, this study designed and fabricated silver-shell-coated gold nanorods (Au@Ag) to synergistically combine the optical advantages of both nanostructures. The LSPR characteristics of Au@Ag nanorods were systematically investigated through FEM modeling (Figure 3). As shown in Figure 3a, the Au@Ag nanorods exhibited dual plasmon resonance peaks at 512 nm and 772 nm. The longitudinal resonance mode showed excellent spectral matching with the 785 nm excitation source, inducing significantly enhanced localized electric fields (Figure 3b). When employed as SERS substrates for detecting 10^−6^ mol/L R6G, the Au@Ag nanorods demonstrated an EF of 5.21 × 10^5^ at the characteristic 612 cm^−1^ peak. The Au@Ag nanorod extends the plasmon resonance wavelength range through its gold core while providing high surface activity via the silver shell. This synergistic combination significantly improves the detection sensitivity of the SERS substrate.

### 3.3. Synergistic Enhancement Mechanisms for GO

The plasma frequency ($\omega_{p}$) of GO could be determined within the Drude model:

(4) $$E\omega_{p}={\left(\frac{4\pi ne^{2}}{\varepsilon_{\infty }m_{e}}\right)}^{1/2}$$

where n represents the electron transport density, $\varepsilon_{\infty }$ denotes the relative permittivity, and $m_{e}$ is the electron mass. The plasmon resonance of GO materials typically occurs in the terahertz range, which cannot be strongly excited by the 785 nm laser used in this study. Therefore, GO does not contribute to the electromagnetic enhancement factor. However, when GO is incorporated into the Au@Ag nanostructure, it could induce interfacial charge transfer, thereby further improving the Raman signal enhancement capability of the substrate.

**Figure 4:**
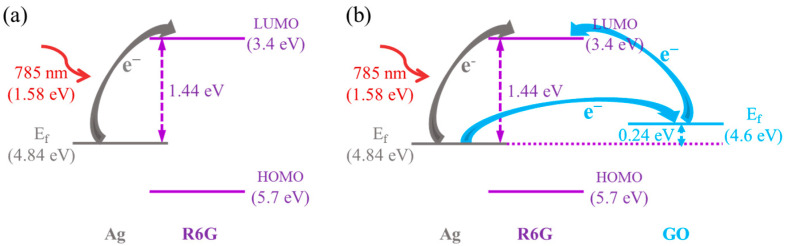
Schematic diagram of charge transfer at the substrate interface. (**a**) Ag and R6G molecule; (**b**) Ag, GO, and R6G molecule.

Taking R6G molecules as an example, the charge transfer process induced by GO materials was analyzed. As shown in Figure 4a, in the isolated Ag-R6G system, the Fermi level of Ag (4.84 eV) and the Lowest unoccupied molecular orbital (LUMO) of R6G (3.4 eV) exhibit an energy gap of approximately 1.44 eV. Under 785 nm laser irradiation (1.58 eV), electrons at the Fermi level of Ag could be excited to the LUMO of R6G, resulting in electron transfer at the Ag–R6G interface. This process leaves Ag positively charged and R6G negatively charged, creating a localized electric field at the interface. With the introduction of GO, as shown in Figure 4b, the Fermi level of GO (4.6 eV) serves as an intermediate energy step under photoexcitation, facilitating electron transfer between Ag and GO, as well as between GO and R6G molecules. The accumulated interfacial charges induce a stronger localized electric field. This charge-transfer-dominated local field could synergistically enhance the Raman signals of adsorbed molecules. The incorporation of GO materials further promotes charge transfer, thereby improving the enhancement sensitivity of the SERS substrate.

**Figure 5:**
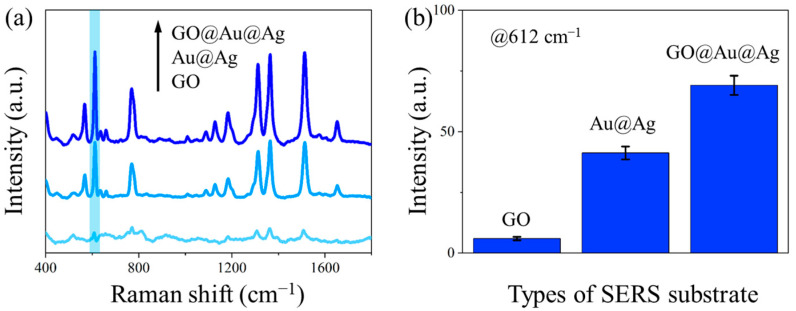
The effect of GO on SERS: (**a**) Raman spectra of R6G enhanced by GO, Au@Ag and GO@Au@Ag, respectively, the characteristic peak of 612 cm^−1^ is marked in blue; (**b**) intensity histograms of the corresponding Raman spectra at 612 cm^−1^ bands.

Figure 5a presents the Raman spectra of R6G molecules obtained from GO, Au@Ag, and GO@Au@Ag nanostructures, respectively, with the corresponding intensity histogram at 612 cm^−1^ shown in Figure 5b. Comparative analysis of the Raman enhancement effects reveals that the incorporation of GO significantly improves the substrate’s enhancement sensitivity. Specifically, the GO@Au@Ag nanostructure demonstrates a 1.68-fold stronger Raman signal enhancement for the target molecule compared to Au@Ag alone. These results demonstrate that (1) the Au@Ag nanostructure exhibits strong Raman enhancement capability, effectively amplifying the Raman signals of analyte molecules; (2) the charge transfer mechanism dominated by GO further improves the enhancement sensitivity of the SERS substrate, synergistically strengthening the Raman signals of analyte molecules.

### 3.4. The Signal Stabilization Effect of NCF

**Figure 6:**
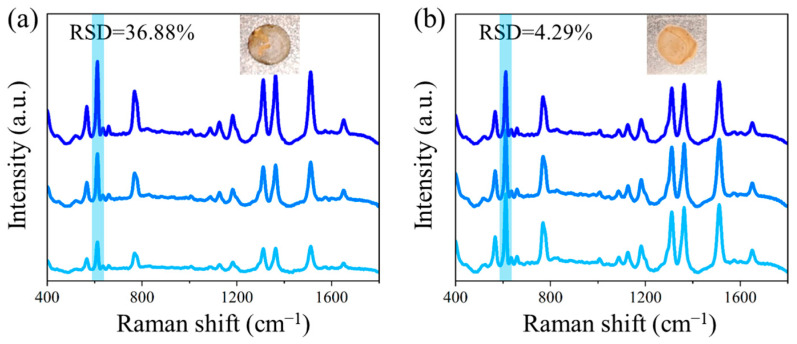
Signal stabilization effect of NCF: uniformity comparison between (**a**) GO/Au@Ag substrate and (**b**) NCF/GO/Au@Ag substrate. The characteristic peaks of 612 cm^−1^ are marked in blue.

The Au@Ag nanorods could be stably attached to the interwoven filaments of NCF, thereby enhancing the stability of SERS system and mitigating the coffee-ring effect. Figure 6 compares the performance of GO/Au@Ag and NCF/GO/Au@Ag substrates in R6G detection with a concentration of 10^−6^ mol/L. The insets show photographs of the respective SERS substrates on the detection platform. In contrast to the apparent coffee-ring effect observed in the GO/Au@Ag substrate (Figure 6a), the NCF-modified substrate demonstrates more uniform nanoparticle distribution (Figure 6b). SERS spectra were randomly acquired from three distinct regions of the circular substrate area: the center (dark blue), midpoint (medium blue), and periphery (light blue). RSD of spectral intensities at the characteristic 612 cm^−1^ peak were calculated to be 36.88% and 4.29% for the control and NCF-modified substrates, respectively. These results demonstrate that NCF incorporation effectively enhances the uniformity of SERS substrates and ensures reliable signal reproducibility.

### 3.5. The Performance of NCF/GO/Au@Ag Substrate

**Figure 7:**
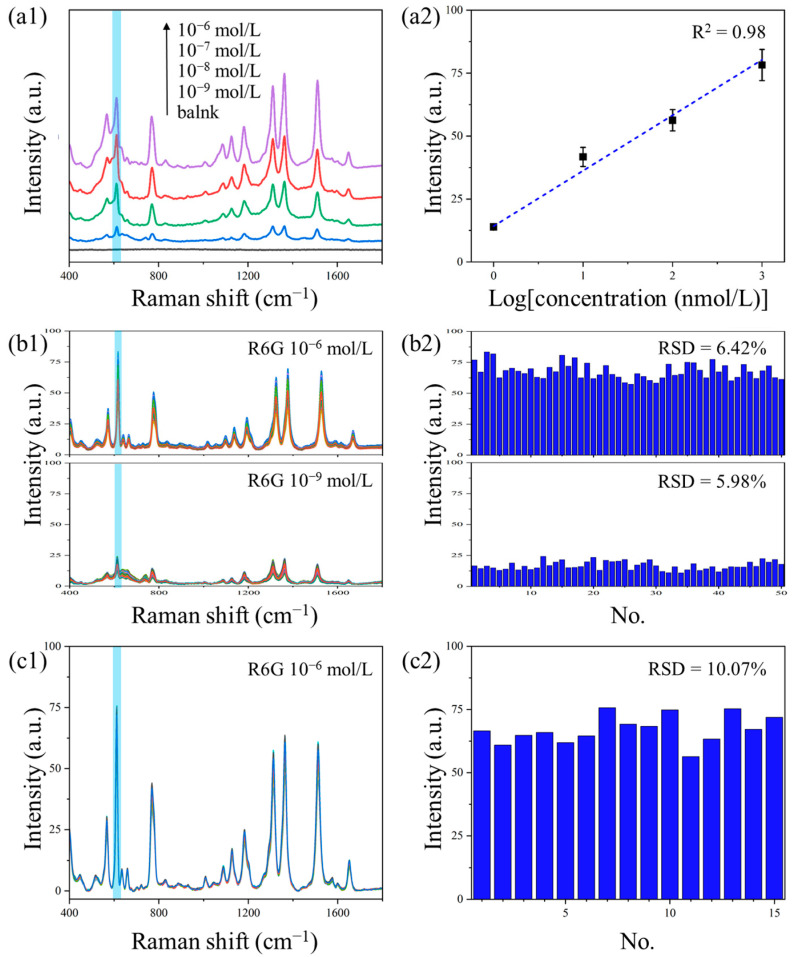
SERS performance of NCF/GO/Au@Ag substrate: (**a1**) sensitivity and (**a2**) calibration curve; (**b1**,**b2**) signal uniformity of R6G at different concentrations; and (**c1**,**c2**) reproducibility between different fabrication batches. The characteristic peaks of 612 cm^−1^ are marked in blue.

The performance of the NCF/GO/Au@Ag substrate was evaluated through enhancement sensitivity, signal uniformity, and batch reproducibility. Figure 7(a1) displays the SERS spectra of R6G at various concentrations collected on the NCF/GO/Au@Ag substrate. The blank substrate showed no characteristic Raman peaks, confirming its non-interference with target molecule detection. Even at an ultra-low concentration of 10^−9^ mol/L, distinct Raman peaks at 612, 1311, 1361, and 1511 cm^−1^ remained detectable, demonstrating the substrate’s exceptional signal enhancement capability for amplifying Raman signals from trace molecules. Notably, as shown in Figure 7(a2), the peak intensity at 612 cm^−1^ exhibited excellent linear correlation (R^2^ = 0.98) with R6G concentrations ranging from 10^−9^ to 10^−6^ mol/L, yielding a calculated LOD of 2.2 × 10^−9^ mol/L.

To evaluate the substrate’s uniformity performance, R6G solutions at two concentrations (10^−6^ mol/L and 10^−9^ mol/L) were selected as probe molecules for testing. As shown in Figure 7(b1,b2), Raman spectra were collected from 50 randomly selected measurement spots on the NCF/GO/Au@Ag substrate, with statistical analysis performed on the intensity distribution of the characteristic peak at 612 cm^−1^. The results demonstrate an RSD of 6.42% and 5.98% for the high and low concentration groups, respectively, indicating satisfactory signal uniformity across the substrate surface. Figure 7(c1) presents the Raman spectra of R6G collected from 15 independently fabricated batches of NCF/GO/Au@Ag substrates, along with the corresponding peak intensities at 612 cm^−1^. The calculated RSD of 10.07% for the 612 cm^−1^ peak intensity in Figure 7(c2) demonstrates excellent batch-to-batch reproducibility. These results confirm that the NCF/GO/Au@Ag substrates exhibit remarkable signal uniformity both within individual substrates and across different production batches, while maintaining facile fabrication repeatability.

### 3.6. Trace Detection of PAHs

**Figure 8:**
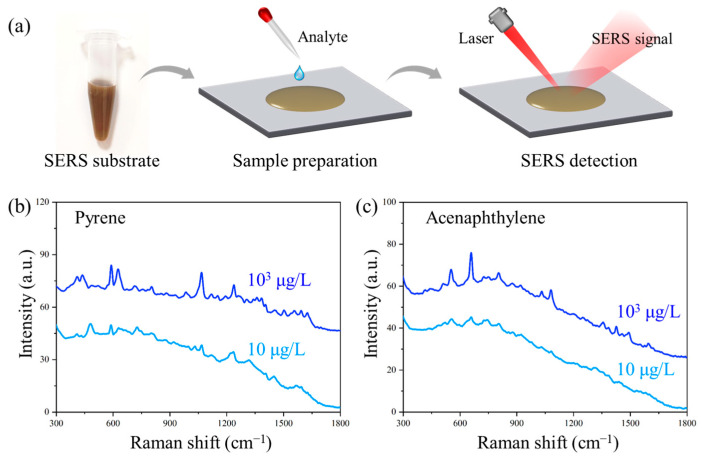
SERS detection of PAHs with NCF/Au@Ag substrate. (**a**) Schematic diagram of SERS detection strategy; SERS spectra of (**b**) pyrene and (**c**) acenaphthylene.

The NCF/GO/Au@Ag substrate was employed for PAH detection. As shown in Figure 8a, the analyte solution was deposited onto the NCF/GO/Au@Ag substrate, followed by Raman signal collection under laser focusing. Each sample was randomly measured and recorded 20 times. The obtained Raman spectra of PAHs are presented in Figure 8b,c. Distinct Raman spectra with clearly identifiable characteristic peaks were successfully acquired even at ultralow concentrations of 10 μg/L for both pyrene and acenaphthylene and demonstrated excellent stability at the concentration of 10^3^ μg/L (Figure S1). These results demonstrate that the NCF/GO/Au@Ag substrate achieves an optimal balance between sensitivity and uniformity, enabling effective signal enhancement for sensitive and stable detection of various molecular analytes.

## 4. Conclusions

The NCF/GO/Au@Ag composite SERS substrate demonstrates satisfactory detection performance, combining sensitivity and stability through synergistic mechanisms. The Au@Ag core–shell nanostructure exhibits strong electromagnetic enhancement due to precise spectral matching between its longitudinal plasmon resonance (772 nm) and the 785 nm excitation wavelength. Concurrently, the incorporated GO enhances charge transfer efficiency, contributing a 1.68-fold improvement in chemical enhancement. Furthermore, the three-dimensional porous framework of NCF effectively immobilizes nanoparticles and significantly improves signal uniformity by 8.6 times, enabling reliable detection of pyrene and acenaphthylene at concentrations as low as 10 μg/L. This work presents an advanced sensing platform for environmental pollutant monitoring, offering both high sensitivity and operational stability, while the innovative material design strategy provides valuable insights for developing next-generation trace detection systems.

## Data Availability

Data available on request.

## Supplementary Materials

The following supporting information can be downloaded at: https://www.mdpi.com/article/10.3390/polym17121716/s1, Figure S1: RSD performance of NCF/GO/Au@Ag substrate for (a) pyrene and (b) acenaphthylene detection with concentration of 10^3^ μg/L.
