# Supplementary material for: Sensitive and Stable NCF/GO/Au@Ag SERS Substrate for Trace Detection of Polycyclic Aromatic Hydrocarbons
Source: Polymers (Basel). 2025 Jun 19;17(12):1716. doi: 10.3390/polym17121716 (PMC12197083; doi:10.3390/polym17121716)
Supplement: Supplementary file 1 [file polymers-17-01716-s001.zip › polymers-3662362-supplementary.pdf]

# Supporting Information

# Sensitive and Stable NCF/GO/Au@Ag SERS Substrate for Trace Detection of Polycyclic Aromatic Hydrocarbons

**Lili Kong [1], Xinna Yu [2], Qifang Sun [3], Meizhen Huang [2], Tianyuan Liu [2,*] and Jie Chen [3,*]**

[1] School of Mathematics, Physics and Statistics, Shanghai University of Engineering Science, Shanghai 201620, China; lilikco@163.com

[2] School of Electronic Information and Electrical Engineering, Shanghai Jiao Tong University, Shanghai 200240, China; xinnayu@sjtu.edu.cn (X.Y); mzhuang@sjtu.edu.cn (M.H.)

[3] College of Information, Mechanical and Electrical Engineering, Shanghai Normal University, Shanghai 200234, China; qifangsun@shnu.edu.cn

* Correspondence: tianyuanl@sjtu.edu.cn (T.L.); jiechen@shnu.edu.cn (J.C.)

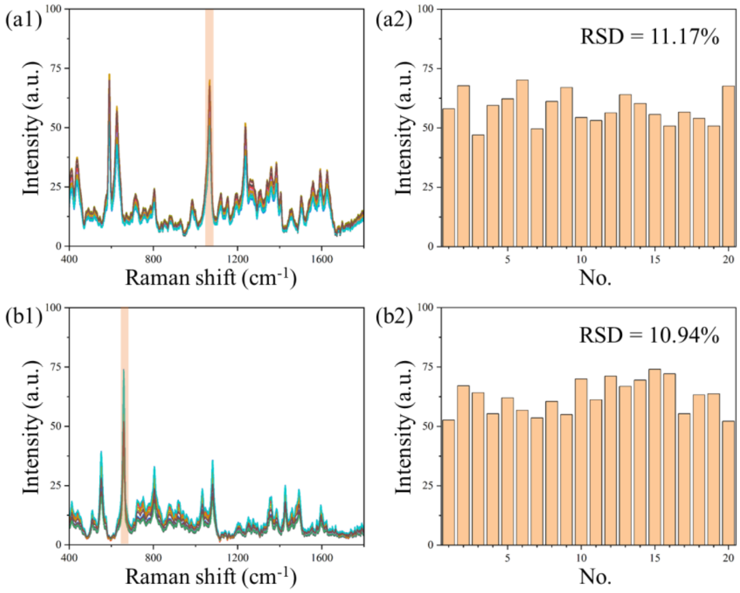


**Figure S1.** RSD performance of NCF/GO/Au@Ag substrate for (a) pyrene and (b) acenaphthylene detection with concentration of $10^3$ μg/L.
